# Association between oxidative stress exposure and colorectal cancer risk in 98,395 participants: results from a prospective study

**DOI:** 10.3389/fnut.2023.1284066

**Published:** 2023-12-15

**Authors:** Haitao Gu, Bo Li, Ling Xiang, Zhiquan Xu, Yunhao Tang, Zhiyong Zhu, Yahui Jiang, Linglong Peng, Hongmei He, Yaxu Wang

**Affiliations:** ^1^Department of Gastrointestinal Surgery, The Second Affiliated Hospital of Chongqing Medical University, Chongqing, China; ^2^Department of Clinical Nutrition, The Second Affiliated Hospital of Chongqing Medical University, Chongqing, China

**Keywords:** oxidative stress exposure, colorectal cancer, epidemiology, sex-specific cohort studies, oxidative balance score

## Abstract

**Background:**

The intricate role of oxidative stress (OS) in colorectal cancer (CRC) initiation is underscored by an imbalance between pro-oxidants and antioxidants. Utilizing the Oxidative Balance Score (OBS) as a metric, this study aims to investigate the association between OS exposure and CRC risk, while also examining potential sex-specific differences in a large U.S. cohort.

**Methods:**

The study included 98,395 adults from the Prostate, Lung, Colorectal, and Ovarian (PLCO) Cancer Screening Trial. To construct the OBS, 14 dietary and lifestyle factors intricately associated with oxidative stress were quantified. A higher OBS value indicated a more favorable oxidative balance pattern or diminished OS exposure. Due to sex-specific differences in OBS, associations were evaluated separately for men and women based on Cox regression analysis. Subgroup analyses were conducted to elucidate potential modifiers.

**Results:**

During 867,963.4 person-years of follow-up, 1,054 CRCs occurred. The mean (SD) age and OBS were 65.52 (5.73) years and 14.09 (3.95) points, respectively. In the fully adjusted Cox model, we observed an inverse association between OBS and CRC incidence in women (HR_*Q*5*vsQ*1_: 0.72; 95% CI: 0.52, 0.99; P for trend = 0.018) but not men. Subgroup analyses revealed the inverse association was more pronounced among women without versus with a family history of CRC (HR_*Q*5__*vsQ*1_: 0.66, 95% CI: 0.47–0.93; P for trend = 0.001; P for interaction = 0.001). The results remained robust after several sensitivity analyses.

**Conclusion:**

Higher OBS was associated with lower CRC risk in women but not men; this inverse association was stronger among women without a family history of CRC. These findings suggest exposure to OS may confer sex-specific CRC risk effects, especially for women without a family history of CRC.

## Introduction

Colorectal cancer (CRC) is a multifaceted disease that ranks third in new cancer cases yet second in cancer mortality. In 2020, over 1.9 million incident CRCs and 935,000 deaths were estimated globally (1). The majority of CRC malignancies arise sporadically, with modifiable lifestyle factors including obesity, physical inactivity, poor diet, alcohol use, and smoking constituting the primary environmental risk factors (2). Numerous investigations have demonstrated that dietary and lifestyle factors play a significant role in the development and progression of CRC, underscoring opportunities for prevention through diet or lifestyle modifications (3, 4). Research into diet and health has shown that nutrients rarely operate in isolation; rather, the combined effects of various dietary and lifestyle factors on CRC risk may be greater than any single element considered individually (5, 6).

Oxidative stress (OS), defined as an imbalance between pro-oxidants and antioxidants favoring the former, is the primary cause of reactive oxygen species and is hypothesized to be involved in colorectal carcinogenesis (7, 8). The oxidative balance score (OBS) allows assessment of an individual’s antioxidant status by accounting for both pro-oxidant and antioxidant components of dietary and lifestyle factors (9–11). As a key metric of cellular metabolism, OBS has been linked to several major human diseases related to health, including cardiovascular disease (9), diabetes (12, 13), and cancer (14). However, current evidence regarding the association between OBS and CRC risk remains inconclusive, with conflicting findings reported. One previous study in 80,063 participants found an increased risk of CRC associated with higher oxidative stress levels (15), while another study using the Health Professionals Follow-up Cohort showed no clear association between overall antioxidant capacity and CRC risk (16). Notably, the components comprising OBS differ between males and females; however, previous investigations have not considered potential sex differences (15). To provide additional epidemiological evidence clarifying these controversial associations while accounting for potential sex disparities, we performed a retrospective analysis stratified by sex in a large U.S. population.

## Materials and methods

### Study design and cohort

The present analysis utilized data from the Prostate, Lung, Colorectal, and Ovarian (PLCO) Cancer Screening Trial. The PLCO trial enrolled 154,887 participants aged 55–74 years at 10 U.S. centers from 1993 to 2001. The trial primarily aimed to evaluate whether screening could reduce mortality for the aforementioned cancers (17). Questionnaires completed by participants included a baseline questionnaire (BQ) capturing demographics and medical history, a dietary history questionnaire (DHQ) using a 137-item food frequency questionnaire (FFQ) to assess dietary intake, and a supplemental questionnaire (SQX). Prior studies have validated the FFQ as a nutritional evaluation tool (18, 19). The PLCO trial was approved by institutional review boards at National Cancer Institute (NCI) and participating centers, and all participants provided informed consent. Specific trial details are published elsewhere (17).

**FIGURE 1:**
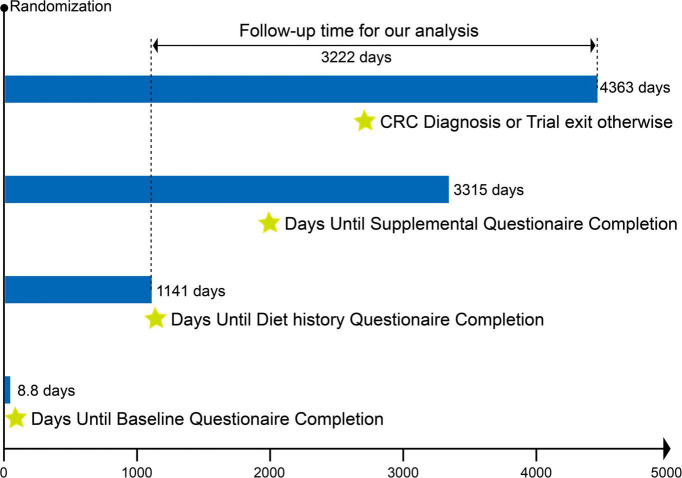
Timeline and follow-up scheme of the study. The mean time from randomization in the PLCO trial to completion of the baseline questionnaire, diet history questionnaire, and supplementary questionnaire was 8.8 days, 1,141 days, and 3,315 days, respectively. The follow-up period for our study was defined as the interval between completion of the diet history questionnaire and the date of CRC diagnosis, death, loss to follow-up, or end of follow-up, whichever came first. The mean follow-up time for our study was 3,222 days.

**FIGURE 2:**
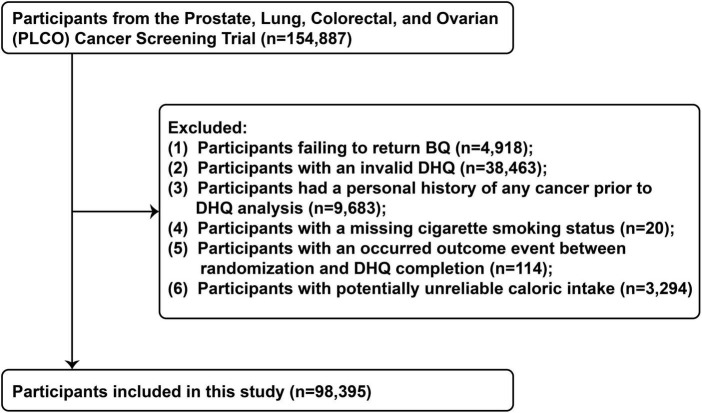
The flow chart of identifying eligible participants. PLCO, Prostate, Lung, Colorectal, and Ovarian; BQ, baseline questionnaire; DHQ, diet history questionnaire.

Our present study aimed to examine the association between OS exposure and CRC risk based on PLCO trial. OS exposure was assessed using the OBS, composed of 14 dietary and lifestyle factors closely related to OS (9). The outcome was incidence of CRC. Follow-up time was defined as the interval between completion of the dietary questionnaire and the date of CRC diagnosis, death, loss to follow-up, or end of follow-up (i.e., December 31, 2009) (Figure 1). Exclusion criteria eliminated the following participants: (I) unreturned BQ (*n* = 4,918); (II) invalid DHQ, defined as ≥ 8 missing responses, extreme caloric intake (gender-specific 1st and 99th percentiles), and DHQ completion date preceding death (*n* = 38,463); (III) personal cancer history before DHQ (*n* = 9,683); (IV) missing smoking status (*n* = 20); (V) outcome event between randomization and DHQ completion (*n* = 114); and (VI) potentially unreliable caloric intake (<800 or > 4,200 kcal/day for males; < 600 or > 3,500 kcal/day for females) (20) (*n* = 3,294). After applying exclusions, 98,395 participants remained eligible (Figure 2).

### Assessment of OBS

We calculated the OBS based on the computational method proposed by Lakkur et al. (9) in 2015. The OBS comprised 14 components selected based on their known associations with OS. In brief, we categorized continuous dietary factors related to antioxidant exposure according to sex-specific tertile cutpoints. Individuals with lower antioxidant exposure (first tertile) for particular dietary antioxidants (e.g., vitamin C, α-carotene, β–carotene, β-Cryptoxanthin, vitamin E, Lutein, Lycopene, selenium) received 0 points, while those with moderate (second tertile) or higher (third tertile) exposure were assigned 1 or 2 points, respectively. Similarly, we classified continuous dietary factors associated with pro-oxidant exposure (polyunsaturated fats and iron) into tertiles, awarding 2 points for low exposure, 1 point for moderate exposure, and 0 points for high exposure. Smoking status was coded as 2 points for never smokers, 1 point for former smokers, and 0 points for current smokers. For aspirin and other NSAIDs use, non-regular use received 0 points, missing responses received 1 point, and regular use received 2 points. Due to sex differences in alcohol intake, we used separate cutoffs to categorize alcohol intake for men versus women. For men, alcohol intake > 14 drinks/week was classified as heavy drinking (0 points), 1–14 drinks/week as moderate drinking (1 point), and no alcohol intake (2 points). For women, alcohol intake > 7 drinks/week was considered heavy drinking (0 points), 1–7 drinks/week as moderate drinking (1 point), and no alcohol intake (2 points). Summing the points for each component produced the total OBS (range 0–28), which we divided into quintiles. A higher OBS indicates a potentially favorable balance between pro-oxidants and antioxidants or lower OS exposure. Supplementary Table 1 summarizes the components comprising the OBS and displays the detailed scoring pattern.

### Identification of CRC cases

In the PLCO trial, all participants were sent an annual health update questionnaire asking them to disclose any new cancer diagnoses, including the cancer site and diagnosis date. Research staff followed-up via phone or email with non-respondents to complete the questionnaire. Separately, death certificates and reports from family members were reviewed to capture additional cancer cases. For self-reported CRC cases, diagnostic verification involved retrieving the medical records to confirm the diagnosis and collect relevant clinical details about CRC. CRC cases were defined using the International Classification of Diseases for Oncology (ICD-O) codes for colon cancer (C18) and rectal cancer (C19–C20).

### Assessment of covariates

Participant demographics, health behaviors, and medical history were obtained at baseline using self-administered questionnaires (including BQ, DHQ and SQX). Demographic variables included race and education level. Lifestyle factors encompassed smoking history and physical activity. Disease history covered family history of CRC, personal history of diabetes, colorectal polyps, colon-related comorbidities, diverticulitis/diverticulosis, and colonoscopy screening. Age at DHQ completion, drinking status and food energy from diet were assessed with the DHQ. Physical activity level was collected through the SQX and defined as the total minutes per week of self-reported moderate to vigorous physical activity. Body mass index (BMI) calculated as weight in kg divided by height squared in m^2^.

### Statistical analysis

For categorical and continuous covariates missing < 5% of data, modal and median imputation was utilized, respectively. Due to a large proportion (up to 25.3%) of missing data, the physical activity level covariate was imputed using multiple imputation methods (21). Further details regarding the imputed datasets are provided in Supplementary Table 2. All statistical analyses were conducted using the imputed datasets.

We utilized Cox proportional hazards regression to evaluate the sex-specific association between the OBS and CRC risk, with follow-up time as the time metric. To test for linear trends, participants were assigned the median OBS value of their quintile, treated as a continuous predictor with the lowest quintile as reference. In the Cox regression analyses, two multivariable Cox models were constructed based on potential confounding variables. Model 1 adjusted for age and race. Model 2 further adjusted for education level, BMI, smoking pack-years, alcohol intake, food energy intake, physical activity level, family history of CRC, history of diabetes, colorectal polyps, colon-related comorbidities, diverticulitis/diverticulosis, and colonoscopy screening.

We conducted stratified analyses across categories of age, BMI, diabetes history, family history of CRC, smoking pack-years, alcohol use, physical activity level, energy intake, and colonoscopy screening history. For continuous subgroup variables, subgroups were defined by dichotomizing at the median based on clinical relevance. Interaction tests were conducted by incorporating OBS-by-subgroup product terms in multivariate Cox models, comparing models with and without the interaction terms.

We performed several sensitivity analyses to evaluate the robustness of the findings: (1) participants with a family history of CRC were excluded, as they may be predisposed to develop CRC. (2) participants with a history of diabetes were excluded, as they may have been required to follow stricter dietary control (22). (3) CRC cases diagnosed within the first 2 and 4 years of follow-up were excluded to minimize reverse causation.

All statistical analyses were performed using R software version 4.3.1. A two-tailed *P*-value < 0.05 was considered statistically significant.

## Results

### Baseline characteristics

The detailed baseline characteristics of the study population across OBS quintiles are presented in Table 1. Among the 98,395 included participants, the mean (standard deviation) OBS was 14.09 (3.95). Participants were categorized into quintiles based on OBS [Quintile 1 (OBS ≤ 10), *n* = 20,125; Quintile 2 (OBS 11–13), *n* = 23,620; Quintile 3 (OBS 14–15), *n* = 17,265; Quintile 4 (OBS 16–18), *n* = 23,042; Quintile 5 (OBS > 18), *n* = 14,343]. Higher quintiles indicated adherence to an antioxidant pattern or lower OS exposure. Compared to the lowest quintile (Quintile 1), those in the highest quintile (Quintile 5) were more likely to be female, have higher educational level, undergo colonoscopy screening, and have greater total energy intake. In the gender baseline characteristics (Supplementary Table 3), compared to males, females had lower BMI, smoking history and intensity, alcohol drinking history and intensity, aspirin use, and higher intakes of dietary antioxidants (e.g., vitamin C, α-carotene, β–carotene, β-cryptoxanthin, vitamin E, lutein). Females also had lower intakes of pro-oxidant dietary components (polyunsaturated fats and iron). Overall, females had higher OBS scores than males.

**TABLE 1:** Baseline characteristics of study population according to quintiles of OBS.

Characteristics	Overall	Quintiles of OBS					*P*-value
		**Quintile 1(≤ 10)**	**Quintile 2(11–13)**	**Quintile 3(14–15)**	**Quintile 4(16–18)**	**Quintile 5(> 18)**	
**Number of participants**	98395	20125	23620	17265	23042	14343	
**OBS**	14.09 ± 3.95	8.54 ± 1.41	12.04 ± 0.81	14.50 ± 0.50	16.94 ± 0.81	20.15 ± 1.24	<0.001
**Age**	65.52 ± 5.73	65.17 ± 5.70	65.45 ± 5.72	65.51 ± 5.73	65.67 ± 5.71	65.88 ± 5.78	<0.001
**Sex**							<0.001
Male	47169 (47.94%)	10754 (53.44%)	11730 (49.66%)	8125 (47.06%)	10453 (45.36%)	6107 (42.58%)	
Female	51226 (52.06%)	9371 (46.56%)	11890 (50.34%)	9140 (52.94%)	12589 (54.64%)	8236 (57.42%)	
**Race**							0.139
White	91159 (92.65%)	18656 (92.70%)	21948 (92.92%)	16015 (92.76%)	21302 (92.45%)	13238 (92.30%)	
Non-white	7236 (7.35%)	1469 (7.30%)	1672 (7.08%)	1250 (7.24%)	1740 (7.55%)	1105 (7.70%)	
**Education level**							<0.001
College below	62550 (63.57%)	14191 (70.51%)	15505 (65.64%)	10794 (62.52%)	13781 (59.81%)	8279 (57.72%)	
College graduate	17348 (17.63%)	3188 (15.84%)	4130 (17.49%)	3136 (18.16%)	4241 (18.41%)	2653 (18.50%)	
Post-graduate	18497 (18.80%)	2746 (13.64%)	3985 (16.87%)	3335 (19.32%)	5020 (21.79%)	3411 (23.78%)	
**Body mass index (kg/m^2^)**	27.20 ± 4.79	27.11 ± 4.59	27.25 ± 4.76	27.18 ± 4.78	27.22 ± 4.85	27.25 ± 5.01	0.021
**Smoking Pack Years**	17.66 ± 26.49	25.39 ± 29.37	19.09 ± 27.11	16.58 ± 25.34	14.59 ± 24.68	10.76 ± 22.18	<0.001
**Drink Alcohol**							<0.001
No	26659 (27.09%)	4417 (21.95%)	6091 (25.79%)	4482 (25.96%)	6654 (28.88%)	5015 (34.96%)	
Yes	71736 (72.91%)	15708 (78.05%)	17529 (74.21%)	12783 (74.04%)	16388 (71.12%)	9328 (65.04%)	
**Family history of CRC**							0.131
No	85990 (87.39%)	17509 (87.00%)	20641 (87.39%)	15108 (87.51%)	20230 (87.80%)	12502 (87.16%)	
Yes/Possibly	12405 (12.61%)	2616 (13.00%)	2979 (12.61%)	2157 (12.49%)	2812 (12.20%)	1841 (12.84%)	
**History of diabetes**							<0.001
No	91932 (93.43%)	19043 (94.62%)	22073 (93.45%)	16148 (93.53%)	21393 (92.84%)	13275 (92.55%)	
Yes	6463 (6.57%)	1082 (5.38%)	1547 (6.55%)	1117 (6.47%)	1649 (7.16%)	1068 (7.45%)	
**History of Colonoscopy Screening**							<0.001
No	51878 (54.46%)	11530 (59.16%)	12777 (56.10%)	8872 (53.09%)	11675 (52.24%)	7024 (50.38%)	
Yes	43388 (45.54%)	7959 (40.84%)	9998 (43.90%)	7840 (46.91%)	10674 (47.76%)	6917 (49.62%)	
**Food Energy from Diet (kcal/day)**	1728.52 ± 657.95	1482.70 ± 578.84	1610.12 ± 615.74	1728.54 ± 635.40	1884.21 ± 668.31	2018.29 ± 664.17	<0.001
**OBS Components**							
Total vitamin C (mg/day)	377.99 ± 387.72	152.96 ± 200.86	285.29 ± 304.72	386.72 ± 363.76	482.05 ± 402.66	668.71 ± 463.52	<0.001
α-Carotene (mcg/day)	845.57 ± 913.09	308.05 ± 241.52	531.45 ± 500.30	790.31 ± 728.45	1169.91 ± 1009.93	1662.56 ± 1241.07	<0.001
Total β–carotene (mcg/day)	4673.45 ± 3850.87	1978.51 ± 1092.36	3163.31 ± 2048.18	4470.62 ± 2862.10	6243.27 ± 3850.30	8663.87 ± 5059.26	<0.001
β-Cryptoxanthin (g/day)	172.16 ± 138.43	83.76 ± 57.99	127.74 ± 88.40	167.46 ± 112.45	219.87 ± 142.50	298.39 ± 179.47	<0.001
Total vitamin E (mg/day)	153.00 ± 176.08	66.32 ± 122.41	124.28 ± 161.28	160.94 ± 176.40	187.65 ± 181.64	256.73 ± 183.62	<0.001
Lutein (mcg/day)	2633.30 ± 2593.67	1207.82 ± 729.95	1817.68 ± 1318.22	2500.71 ± 2002.23	3510.53 ± 2921.49	4726.89 ± 3796.97	< 0.001
Lycopene (mcg/day)	6447.77 ± 6825.38	3687.23 ± 2552.38	4933.19 ± 3762.14	6188.45 ± 5073.10	8038.54 ± 8457.18	10571.94 ± 10278.19	<0.001
Total selenium (mcg/day)	89.38 ± 39.64	71.62 ± 31.23	81.17 ± 34.88	89.66 ± 37.92	99.65 ± 40.94	110.98 ± 42.40	<0.001
PUFA (g/day)	14.05 ± 7.15	12.21 ± 6.18	13.28 ± 6.74	14.17 ± 7.00	15.33 ± 7.60	15.72 ± 7.72	<0.001
Total iron (mg/day)	23.74 ± 11.40	18.36 ± 10.17	21.74 ± 10.44	24.31 ± 10.64	26.90 ± 11.10	28.84 ± 11.97	<0.001
Smoking history							<0.001
never smoker	47196 (47.97%)	6577 (32.68%)	10430 (44.16%)	8465 (49.03%)	12435 (53.97%)	9289 (64.76%)	
current smoker	8987 (9.13%)	3883 (19.29%)	2354 (9.97%)	1241 (7.19%)	1175 (5.10%)	334 (2.33%)	
former smoker	42212 (42.90%)	9665 (48.02%)	10836 (45.88%)	7559 (43.78%)	9432 (40.93%)	4720 (32.91%)	
Aspirin							<0.001
never	51787 (52.63%)	14638 (72.74%)	13387 (56.68%)	9140 (52.94%)	10605 (46.02%)	4017 (28.01%)	
regular user	46190 (46.94%)	5392 (26.79%)	10120 (42.85%)	8050 (46.63%)	12350 (53.60%)	10278 (71.66%)	
missing	418 (0.42%)	95 (0.47%)	113 (0.48%)	75 (0.43%)	87 (0.38%)	48 (0.33%)	
Other NSAIDs							<0.001
never	68070 (69.18%)	15212 (75.59%)	16832 (71.26%)	12081 (69.97%)	15681 (68.05%)	8264 (57.62%)	
regular user	3922 (3.99%)	332 (1.65%)	727 (3.08%)	668 (3.87%)	1042 (4.52%)	1153 (8.04%)	
missing	26403 (26.83%)	4581 (22.76%)	6061 (25.66%)	4516 (26.16%)	6319 (27.42%)	4926 (34.34%)	
Alcohol (drinks/week)	0.65 ± 1.41	0.93 ± 1.85	0.70 ± 1.49	0.64 ± 1.32	0.55 ± 1.19	0.35 ± 0.83	<0.001

Values are means (standard deviation) for continuous variables and percentages for categorical variables. Group comparisons of continuous variables utilized analysis of variance (ANOVA). Categorical variables employed chi-squared tests to assess differences across quartiles.

### Association between OBS and CRC incidence

Over a mean (SD) follow-up time of 8.82 (1.95) years totaling 867,963.4 person-years, 1054 CRC cases (571 female and 483 male) were documented among 98,395 participants (51,226 women and 47,169 men), reflecting an overall CRC incidence rate of 12 cases per 10,000 person-years, with incidence rates of 10.6 and 13.9 cases per 10,000 person-years in women and men, respectively. Compared to women, the CRC incidence rate in men was 31.1% higher (95% CI: 30.1% to 32.2%). In this study, we identified an inverse association between OBS and CRC incidence in women but not men. In women, the unadjusted model showed a lower CRC risk for those in the highest OBS quintile compared to the lowest quintile (HR_*Q*5*vs*_._*Q*1_: 0.66; 95% CI: 0.49, 0.89; P for trend = 0.001) (Table 2). This inverse association persisted after full adjustment for potential confounders (HR_*Q*5*vsQ*1_: 0.72; 95% CI: 0.52, 0.99; P for trend = 0.018) (Table 2). However, no significant association was observed in men in either the crude or fully adjusted models (Table 2).

**TABLE 2:** Association of OBS with the risk of colorectal cancer by sex in the PLCO cohort^a^.

Quintiles of OBS score	No. of Participants	No. of Cases	Person-years	Hazard ratio (95% confidence interval)		
				**Unadjusted**	**Model 1 ^b^**	**Model 2 ^c^**
**Male**						
Quintile 1 (≤ 10)	10219	131	88415.35	1.00 (reference)	1.00 (reference)	1.00 (reference)
Quintile 2 (11–13)	11389	125	99258.22	0.85(0.66,1.09)	0.84(0.65,1.07)	0.89(0.69,1.13)
Quintile 3 (14–15)	8117	100	71010.12	0.95(0.73,1.23)	0.93(0.72,1.21)	1.03(0.79,1.34)
Quintile 4 (16–18)	10807	127	94659.44	0.90(0.71,1.16)	0.88(0.69,1.12)	1.01(0.78,1.31)
Quintile 5 (> 18)	6637	88	58028.89	1.02(0.78,1.34)	0.98(0.74,1.28)	1.20(0.89,1.61)
*P* for trend				0.863	0.865	0.199
**Female**						
Quintile 1 (≤ 11)	13833	155	122621.94	1.00 (reference)	1.00 (reference)	1.00 (reference)
Quintile 2 (12–13)	8307	92	73672.52	0.99(0.76,1.28)	0.97(0.75,1.26)	1.01(0.78,1.31)
Quintile 3 (14–15)	9057	71	80816.36	0.70(0.53,0.92)	0.68(0.51,0.90)	0.72(0.54,0.96)
Quintile 4 (16–18)	12118	106	108488.79	0.77(0.61,0.99)	0.76(0.59,0.97)	0.83(0.64,1.08)
Quintile 5 (> 18)	7911	59	70991.78	0.66(0.49,0.89)	0.64(0.47,0.86)	0.72(0.52,0.99)
*P* for trend				**0.001**	** < 0.001**	**0.018**

*^a^*Hazard ratio was calculated using Cox proportional hazard regression models, P-values were calculated from significance testing for the underlying linear trend in Cox models.

*^b^*Adjusted for age (years) and race (white, non-white).

*^c^*Adjusted for model 1 plus educational level (college below, college graduate, post-graduate), body mass index (kg/m^2^), family history of colorectal cancer (no, yes/possibly), pack-years (continuous), drinker (no, yes), history of diabetes (no, yes), physical activity (min/week), history of colon screen (no, yes), history of colorectal polyps (no, yes), history of colon related co-morbidity (no, yes), history of diverticulitis or diverticulosis (no, yes) and food energy from diet (kcal/day).

### Additional analyses

As shown in Table 3, subgroup analyses revealed no modification by age, BMI, history of diabetes, pack-years of smoking, drinking status, physical activity level, food energy from diet or history of colonoscopy screening on the OBS-CRC risk association in women (all P for interaction > 0.05). Interestingly, the inverse OBS-CRC risk association was more pronounced among women without a family history of CRC (HR_*Q*5*vsQ*1_: 0.66, 95% CI: 0.47–0.93; P for trend = 0.001), with a significant interaction by family history of CRC (P for interaction = 0.001). In sensitivity analyses excluding those with family history of CRC, history of diabetes, or early follow-up, the inverse OBS-CRC risk association persisted among women (Table 4).

**TABLE 3:** Subgroup analyses on the association of OBS with the risk of colorectal cancer in females^a^.

Subgroup variable	No. of cases	Person-years	Hazard Ratio (95% Confidence Interval) by OBS ^b^					*P*_*trend*_	*P*_*interaction*_
			**Quintile 1 (≤ 11)**	**Quintile 2 (12–13)**	**Quintile 3 (14–15)**	**Quintile 4 (16–18)**	**Quintile 5 (> 18)**		
**Age (years)**									0.089
≤ 65	190	246467.46	1.00(reference)	1.04 (0.71, 1.54)	0.55(0.34, 0.89)	0.52(0.33, 0.82)	0.65(0.39, 1.07)	0.004	
> 65	293	210123.93	1.00(reference)	0.98(0.69, 1.39)	0.85(0.59, 1.22)	1.09(0.78, 1.51)	0.78(0.51, 1.20)	0.531	
**BMI(kg/m^2^)**									0.692
≤ 30	360	352305.45	1.00(reference)	0.93(0.69, 1.26)	0.63(0.45, 0.90)	0.80(0.59, 1.08)	0.69(0.47, 1.00)	0.021	
> 30	123	104285.94	1.00(reference)	1.27(0.76, 2.11)	1.03(0.60, 1.78)	0.95(0.55, 1.63)	0.86(0.45, 1.65)	0.486	
**History of Diabetes**									0.519
No	438	433569.64	1.00(reference)	0.93(0.71, 1.23)	0.72(0.54, 0.97)	0.82(0.62, 1.07)	0.70(0.49, 0.98)	0.019	
Yes	45	23021.75	1.00(reference)	1.89(0.85, 4.19)	0.67(0.23, 1.95)	0.99(0.40, 2.44)	0.91(0.32, 2.64)	0.592	
**Family History of Colorectal Cancer**									0.001
No	419	397607.4	1.00(reference)	0.95(0.72, 1.25)	0.64(0.47, 0.87)	0.66(0.49, 0.88)	0.66(0.47, 0.93)	0.001	
Yes/possibly	64	58984.02	1.00(reference)	1.82(0.76, 4.40)	1.87(0.78, 4.45)	3.47(1.61, 7.48)	1.53(0.55, 4.26)	0.040	
**Smoking Pack Years**									0.162
< = median	272	267605.57	1.00(reference)	1.14(0.80, 1.61)	0.76(0.52, 1.11)	0.76(0.53, 1.09)	0.65(0.42, 0.99)	0.014	
>median	211	188985.82	1.00(reference)	0.84(0.56, 1.26)	0.67(0.43, 1.04)	0.94(0.64, 1.38)	0.93(0.56, 1.53)	0.612	
**Drink Alcohol**									0.192
no	151	138737.56	1.00(reference)	1.31(0.83, 2.07)	0.99(0.61, 1.63)	0.86(0.53, 1.40)	0.62(0.34, 1.14)	0.094	
yes	332	317853.83	1.00(reference)	0.89(0.64, 1.22)	0.62(0.43, 0.88)	0.82(0.60, 1.13)	0.78(0.53, 1.14)	0.120	
**Physical Activity Level (min/week)**									0.603
< = median	287	229171.81	1.00(reference)	1.12(0.81, 1.54)	0.74(0.51, 1.07)	0.87(0.62, 1.22)	0.61(0.38, 0.98)	0.056	
>median	196	227419.58	1.00(reference)	0.79(0.51, 1.24)	0.66(0.42, 1.04)	0.74(0.49, 1.12)	0.78(0.49, 1.24)	0.207	
**Food Energy from Diet (kcal/day)**									0.947
< = median	261	228187.90	1.00(reference)	1.05(0.76, 1.45)	0.73(0.50, 1.05)	0.75(0.52, 1.09)	0.67(0.39, 1.14)	0.030	
>median	222	228403.49	1.00(reference)	0.91(0.58, 1.42)	0.69(0.44, 1.08)	0.85(0.58, 1.25)	0.72(0.46, 1.10)	0.152	
**History of Colonoscopy Screening**									0.458
no	265	243502.74	1.00(reference)	1.08(0.77, 1.52)	0.78(0.54, 1.14)	0.74(0.51, 1.07)	0.82(0.53, 1.26)	0.139	
yes	218	213088.65	1.00(reference)	0.92(0.61, 1.37)	0.65(0.42, 1.00)	0.92(0.63, 1.34)	0.61(0.37, 1.00)	0.080	

*^a^*Hazard ratio was calculated using Cox proportional hazard regression models, P trend was calculated from significance testing for the underlying linear trend in Cox models, P interaction for likelihood ratio tests was calculated from significance testing of interaction terms in Cox models.

*^b^*Hazard ratios were adjusted for age (years), race (white, non-white), educational level (college below, college graduate, post-graduate), body mass index (kg/m2), family history of colorectal cancer (no, yes/possibly), pack-years (continuous), drinker (no, yes), history of diabetes (no, yes), physical activity (min/week), history of colon screen (no, yes), history of colorectal polyps (no, yes), history of colon related co-morbidity (no, yes), history of diverticulitis or diverticulosis (no, yes) and food energy from diet (kcal/day).

**TABLE 4:** Sensitivity analyses on the association of OBS with the risk of colorectal cancer in female ^a^.

Categories	No. of Participants	No. of Cases	Hazard Ratio (95% Confidence Interval) by OBS ^b^					*P*_*trend*_
			**Quintile 1 (≤ 11)**	**Quintile 2 (12–13)**	**Quintile 3 (14–15)**	**Quintile 4 (16–18)**	**Quintile 5 (> 18)**	
Excluded participants with family history of colorectal cancer ^c^	44597	419	1.00 (reference)	0.95 (0.72,1.25)	0.64 (0.47,0.87)	0.66 (0.49,0.88)	0.66 (0.47,0.93)	0.001
Excluded participants with a history of diabetes ^d^	48474	438	1.00 (reference)	0.93 (0.71,1.23)	0.72 (0.54,0.97)	0.82 (0.62,1.07)	0.70 (0.49,0.98)	0.019
Excluded cases observed within the first 2 years of follow-up	51113	370	1.00 (reference)	0.89 (0.66,1.20)	0.66 (0.48,0.92)	0.79 (0.59,1.07)	0.64 (0.44,0.94)	0.011
Excluded cases observed within the first 4 years of follow-up	50995	252	1.00 (reference)	1.04 (0.73,1.49)	0.69 (0.46,1.04)	0.90 (0.63,1.29)	0.71 (0.45,1.12)	0.041

*^a^*Hazard ratio was calculated using Cox proportional hazard regression models, P trend was calculated from significance testing for the underlying linear trend in Cox models.

*^b^*Hazard ratios were adjusted for age (years), race (white, non-white), educational level (college below, college graduate, post-graduate), body mass index (kg/m2), family history of colorectal cancer (no, yes/possibly), pack-years (continuous), drinker (no, yes), history of diabetes (no, yes), physical activity (min/week), history of colon screen (no, yes), history of colorectal polyps (no, yes), history of colon related co-morbidity (no, yes), history of diverticulitis or diverticulosis (no, yes) and food energy from diet (kcal/day).

*^c^*Hazard ratio was not adjusted for history of colorectal cancer.

*^d^*Hazard ratio was not adjusted for history of diabetes.

### Discussion

In this study, higher OBS were associated with a lower risk of CRC in women. However, no significant association was observed in men. Subgroup analyses showed that the inverse association was stronger in women with no family history compared to those with a family history of CRC. The inverse association remained robust in sensitivity analyses excluding participants with potential confounding characteristics, lending strength to the conclusions.

CRC has been demonstrated to be closely related to dietary and lifestyle factors. For example, adherence to the Mediterranean diet (MD) and Dietary Approaches to Stop Hypertension (DASH) dietary patterns have been associated with lower incidence of colorectal cancer in several studies (23, 24). The OBS constructed in this study incorporated 14 dietary and lifestyle indicators with established links to OS exposures. While the OBS has been linked with several major chronic human diseases related to health, including cardiovascular disease (9), diabetes (12, 13), and cancer (14). It should be noted that although prior studies have explored OBS in relation to CRC (15), the overall linkage between OS exposure and CRC risk remains ambiguous with inconsistent literature findings (16). The pathogenesis of CRC is intimately connected with factors that heighten OS and impair antioxidant defenses. For instance, lifestyle factors of OBS like smoking and alcohol enlarge reactive oxygen species production, whereas reduced antioxidant enzyme activity and DNA repair capacity attenuate antioxidant protection (25, 26). Conversely, sufficient intakes of antioxidant nutrients such as vitamins E and carotenoids can remove excess reactive oxygen species, boost antioxidant enzyme activity, safeguard DNA from oxidative damage, and thus mitigate CRC occurrence (27–30). In addition, it has been demonstrated that reactive oxygen species (ROS) generated by OS can disrupt critical cellular functions by interacting with cellular macromolecules, including proteins, nucleic acids, and lipids (31). For instance, oxidative damage to DNA may result in base oxidation, single- and double-strand breaks, or the creation of non-basic sites (32). Furthermore, unrepaired oxidative DNA damage enhances the risk of mutagenesis. If these mutations occur in genes imperative for regulating cell growth, such as tumor suppressor genes and proto-oncogenes, they may engender CRC (33). The body’s response to injury of intestinal mucosal cells exposed to oxidative stress is inflammation. Repeated exposure to inflammatory sites can elicit chronic inflammation and activation of autoimmune processes (34). Inflammation instigates epigenetic alterations that promote colorectal carcinogenesis through increased production of growth factors and proinflammatory cytokines (35). Animal and clinical investigations have delineated the primary mechanism by which free radicals contribute to colorectal carcinogenesis; specifically, free radicals intercede in inflammation and carcinogenesis via the transcription factor NRF2 (36–38). Therefore, OBS as an integrative indicator of *in vivo* redox balance exhibits clear biological relevance to CRC risk, although the exact associations and gender differences warrant further investigation.

A unique finding of this study was the effect modification by sex on the association between OBS and CRC risk. The potential reasons may relate to the following points: (I) Several studies indicate lower NADPH oxidase activity and function in females, attributable to direct estrogen-mediated reduction of NADPH oxidase activity as well as lower expression of the essential assembly factor p47 and superoxide production, independent of estrogen effects, culminating in lower superoxide levels in females with lower oxidative stress (39–42). (II) Clinical and experimental studies have indicated that women have stronger antioxidant potential than men (43). This may be because estrogen has antioxidant qualities, making women less vulnerable to oxidative stress (44). (III) In our present analysis, the CRC incidence rate is lower in women and the gender baseline characteristics showed that relative to men, women often adopt healthier lifestyles, such as limited smoking and alcohol consumption, that may reduce oxidative damage and inflammation (45). Additionally, Supplementary Table 3 also showed that women had higher intakes of antioxidant nutrients and higher OBS score, which may minimize oxidative damage and preserve oxidative balance, thereby lowering CRC risk (46).

This study has several notable strengths. It was a well-designed observational study in a large population, and the extensive follow-up period of up to 8 years allowed sufficient time for outcome events to occur. Moreover, we extensively adjusted for potential demographic, lifestyle, and disease history confounders, thereby minimizing residual confounding of the observed associations. Importantly, we identified a gender difference in the association between OBS and colorectal cancer risk. Furthermore, the inverse association demonstrated good robustness across multiple sensitivity analyses.

Some limitations should be acknowledged. Firstly, multiple variants of the OBS scoring system have been developed in prior studies (11, 47, 48). The present study developed an OBS scoring system using the framework proposed by Lakkur et al. (9), which integrated 14 dietary and lifestyle factors into the score calculation. Given the controversial role of PUFAs, aspirin, and NSAIDs on OS (49, 50), we reconstructed the OBS score after removing these 3 components. This reconstructed score was associated with the occurrence of CRC in the unadjusted and demographic-adjusted models, but no statistically significant association was observed in the fully adjusted model (Supplementary Table 4). Therefore, caution must be taken when examining the relationship between OS and CRC, as differing OBS scoring systems may lead to disparate results. In addition, incorporating direct biomarkers of OS, such as markers of DNA damage or lipid peroxidation, could have provided more objective measures of OBS. Unfortunately, such biomarkers were not available in the database used for this analysis. Secondly, dietary data was solely gathered at the baseline. Any shifts in dietary habits during the follow-up period could introduce non-differential misclassification bias. Thirdly, our study cohort exclusively comprised American adults aged 55–74. Therefore, the generalizability of the conclusions remains subject to further investigation. Fourthly, as detailed genetic data and important blood markers (e.g., hormones and estrogen) were not available in the PLCO cohort, which limits our ability to explore the impacts of genetic predispositions, molecular subtypes, and familial predispositions on the association between OBS and CRC risk as well as its gender difference. This is an important limitation of our study. In future research, we will utilize data from databases with genomic information and biological blood markers (e.g., UK Biobank) to further explore these factors in OBS-related CRC development and distinguish the associations in different sex and CRC subtypes. Finally, while this large, observational study with lengthy follow-up identified an association between OBS and CRC in female, the lack of genomic data is a limitation to establish causal relationships through Mendelian randomization approaches.

## Conclusion

In this study of U.S. adults, higher OBS were associated with lower CRC risk among women but not men. This suggests that adherence to an antioxidant diet and lifestyle pattern may aid in CRC prevention, particularly for women without a family history of CRC. Further research is warranted to confirm these findings and should consider potential sex-specific mechanisms.

## Data availability statement

Publicly available datasets were analyzed in this study. This data can be found here: https://cdas.cancer.gov/.

## Ethics statement

The studies involving human participants were reviewed and approved by the Institutional Review Board of the National Cancer Institute. The patients/participants provided their written informed consent to participate in the PLCO study.

## Author contributions

LX: Conceptualization, Formal analysis, Resources, Visualization, Writing-original draft. BL: Writing-original draft, Data curation, Methodology. HG: Methodology, Funding acquisition, Writing-review and editing. ZX: Methodology, Writing-review and editing. YT: Methodology, Writing-review and editing. ZZ: Methodology, Writing-review and editing. YJ: Methodology, Writing-review and editing. LP: Methodology, Formal analysis, Funding acquisition, Writing-review and editing. HH: Writing-review and editing, Conceptualization, Supervision. YW: Conceptualization, Supervision, Writing-review and editing, Funding acquisition.
