# Supplementary material for: Association between oxidative stress exposure and colorectal cancer risk in 98,395 participants: results from a prospective study
Source: Front Nutr. 2023 Dec 15;10:1284066. doi: 10.3389/fnut.2023.1284066 (PMC10773815; doi:10.3389/fnut.2023.1284066)
Supplement: Supplementary file 1 [file Data_Sheet_1.PDF]

Supplementary Table 1. Oxidative balance score (OBS) point assignment scheme

OBS components		Score assignment scheme	
Total vitamin C [a]	0 = low (1st tertile)	1 = medium (2nd tertile)	2 = high (3rd tertile)
α-Carotene	0 = low (1st tertile)	1 = medium (2nd tertile)	2 = high (3rd tertile)
Total β-carotene [a]	0 = low (1st tertile)	1 = medium (2nd tertile)	2 = high (3rd tertile)
β-Cryptoxanthin	0 = low (1st tertile)	1 = medium (2nd tertile)	2 = high (3rd tertile)
Total vitamin E [a]	0 = low (1st tertile)	1 = medium (2nd tertile)	2 = high (3rd tertile)
Lutein	0 = low (1st tertile)	1 = medium (2nd tertile)	2 = high (3rd tertile)
Lycopene	0 = low (1st tertile)	1 = medium (2nd tertile)	2 = high (3rd tertile)
Total selenium [a]	0 = low (1st tertile)	1 = medium (2nd tertile)	2 = high (3rd tertile)
PUFA	0 = high (3rd tertile)	1 = medium (2nd tertile)	2 = low (1st tertile)
Total iron [a]	0 = high (3rd tertile)	1 = medium (2nd tertile)	2 = low (1st tertile)
Smoking history	0 = current smoker	1 = former smoker	2 = never smoker
Aspirin	0 = never	1 = missing	2 = regular user
Other NSAIDs	0 = never	1 = missing	2 = regular user
Alcohol			
Male	0 = >14 drinks/week	1 = 1-14 drinks/week	2 = none
Female	0 = >7 drinks/week	1 = 1-7 drinks/week	2 = none

Abbreviations: NSAID = nonsteroidal anti-inflammatory drug (not including aspirin); PUFA = polyunsaturated fatty acids

a Total intake = dietary plus supplement intakes; inclusion of supplemental intake based on the availability of supplemental intake information

Supplementary Table 2. Distribution of variables with missing data before and after imputation

Variable	Before imputation	After imputation	No. (%) with missing data
Educational level			178(0.18%)
College below	62372 (63.50%)	62550 (63.57%)	
College graduate	17348 (17.66%)	17348 (17.63%)	
Postgraduate	18497 (18.83%)	18497 (18.80%)	
Body mass index (kg/m^2)	27.21±4.82	27.20±4.79	1283(1.30%)
Smoking pack years	17.66±26.49	17.49±26.40	1084(1.10%)
Family history of colorectal cancer			745(0.76%)
No	85245 (87.30%)	85990 (87.39%)	
Yes/Possibly	12405 (12.70%)	12405 (12.61%)	
History of diabetes			511(0.52%)
No	91421 (93.40%)	91932 (93.43%)	
Yes	6463 (6.60%)	6463 (6.57%)	
Physical activity (min/week)	125.15±123.32	122.58±108.67	24896(25.30%)
History of Colonoscopy Screening			3129(3.18%)
No	51878 (54.46%)	55007(55.90%)	
Yes	43388 (45.54%)	43388(44.10%)	

Supplementary Table 3. Baseline characteristics of study population according to sex

Characteristics	All participants	Male	Female	*P*-value
**Number**	98395	47169	51226	
**OBS**	14.09±3.95	13.78±3.94	14.37±3.93	<0.001
**Age**	65.52±5.73	65.78±5.74	65.27±5.71	<0.001
**Race**				0.336
White	91159 (92.65%)	43740 (92.73%)	47419 (92.57%)	
Non-white	7236 (7.35%)	3429 (7.27%)	3807 (7.43%)	
**Education level**				<0.001
College below	62550 (63.57%)	27152 (57.56%)	35398 (69.10%)	
College graduate	17348 (17.63%)	9291 (19.70%)	8057 (15.73%)	
Postgraduate	18497 (18.80%)	10726 (22.74%)	7771 (15.17%)	
**Body mass index (kg/m^2)**	27.20±4.79	27.47±4.04	26.96±5.37	<0.001
**Food Energy from Diet (kcal/day)**	1728.52±657.95	1970.04±694.48	1506.13±533.16	<0.001
**Smoking Pack Years**	17.49±26.40	23.26±29.94	12.18±21.31	<0.001
**Drink Alcohol**				<0.001
No	26659 (27.09%)	10966 (23.25%)	15693 (30.63%)	
Yes	71736 (72.91%)	36203 (76.75%)	35533 (69.37%)	
**History of diabetes**				<0.001
No	91932 (93.43%)	43458 (92.13%)	48474 (94.63%)	
Yes	6463 (6.57%)	3711 (7.87%)	2752 (5.37%)	
**Family history of colorectal cancer**				0.001
No	85990 (87.39%)	41393 (87.75%)	44597 (87.06%)	
Yes/Possibly	12405 (12.61%)	5776 (12.25%)	6629 (12.94%)	
**History of Colonoscopy Screening**				<0.001
No	55007 (55.90%)	27466 (58.23%)	27541 (53.76%)	
Yes	43388 (44.10%)	19703 (41.77%)	23685 (46.24%)	
**Total vitamin C (mg/day)**	377.99±387.72	354.48±381.62	399.64±392.02	<0.001
**α-Carotene (mcg/day)**	845.57±913.09	779.94±852.23	906.01±961.79	<0.001
**Total β–carotene (mcg/day)**	4673.45±3850.87	4362.18±3744.99	4960.06±3924.12	<0.001
**β-Cryptoxanthin (g/day)**	172.16±138.43	168.98±136.28	175.09±140.33	<0.001
**Total vitamin E (mg/day)**	153.00±176.08	136.75±172.83	167.97±177.72	<0.001
**Lutein (mcg/day)**	2633.30±2593.67	2606.12±2564.00	2658.32±2620.47	0.002
**Lycopene (mcg/day)**	6447.77±6825.38	7242.26±7530.37	5716.21±6012.60	<0.001
**Total selenium (mcg/day)**	89.38±39.64	102.87±42.36	76.96±32.32	<0.001
**PUFA (g/day)**	14.05±7.15	15.61±7.61	12.62±6.38	<0.001
**Total iron (mg/day)**	23.74±11.40	24.31±11.64	23.21±11.15	<0.001
**Smoking history**				<0.001
never smoker	47196 (47.97%)	17850 (37.84%)	29346 (57.29%)	
current smoker	8987 (9.13%)	4691 (9.95%)	4296 (8.39%)	
former smoker	42212 (42.90%)	24628 (52.21%)	17584 (34.33%)	
**Aspirin**				<0.001
never	51787 (52.63%)	22398 (47.48%)	29389 (57.37%)	
regular user	46190 (46.94%)	24462 (51.86%)	21728 (42.42%)	
missing	418 (0.42%)	309 (0.66%)	109 (0.21%)	
**Other NSAIDs**				<0.001
never	68070 (69.18%)	32442 (68.78%)	35628 (69.55%)	
regular user	3922 (3.99%)	1374 (2.91%)	2548 (4.97%)	
missing	26403 (26.83%)	13353 (28.31%)	13050 (25.48%)	
**Alcohol(drinks/week)**	0.65±1.41	0.95±1.80	0.37±0.83	<0.001

Values are means (standard deviation) for continuous variables and percentages for categorical variables. Group comparisons of continuous variables utilized Student's t-tests. Categorical variables employed chi-squared tests to assess differences across quartiles.

Supplementary Table 4. Association of reconstructed OBS with the risk of colorectal cancer in women [a]

Quintiles of OBS score	No. of Participants	No. of Cases	Person-years	Hazard ratio (95% confidence interval)		
				Unadjusted	Model 1 [b]	Model 2 [c]
Quintile 1 (≤8)	11993	129	103989.00	1.00 (reference)	1.00 (reference)	1.00 (reference)
Quintile 2 (9-11)	12154	135	106857.34	1.02 (0.80, 1.30)	1.01 (0.80, 1.29)	1.04 (0.82, 1.33)
Quintile 3 (12-13)	8630	81	76253.56	0.86 (0.65, 1.13)	0.85 (0.64, 1.12)	0.89 (0.67, 1.18)
Quintile 4 (14-15)	8298	77	73924.28	0.84 (0.63, 1.12)	0.83 (0.62, 1.10)	0.87 (0.65, 1.17)
Quintile 5 (>15)	10151	92	91150.79	0.82 (0.62, 1.07)	0.81 (0.62, 1.05)	0.87 (0.64, 1.18)
P for trend				0.051	0.038	0.195

a: The reconstructed OBS refers to the original OBS scoring system with PUFAs, aspirin, and NSAIDs removed as components. Hazard ratio was calculated using Cox proportional hazard regression models, P values were calculated from significance testing for the underlying linear trend in Cox models.

b: Adjusted for age (years) and race (white, non-white).

c: Adjusted for model 1 plus educational level (college below, college graduate, postgraduate), body mass index (kg/m^2), family history of colorectal cancer (no, yes/possibly), pack-years (continuous), drinker (no, yes), history of diabetes (no, yes), physical activity (min/week), history of colon screen (no, yes), history of colorectal polyps (no, yes), history of colon related co-morbidity (no, yes), history of diverticulitis or diverticulosis (no, yes) and food energy from diet (kcal/day).
